# Assessment of the Shear Bond Strength between Nanofilled Composite Bonded to Glass-ionomer Cement Using Self-etch Adhesive with Different pHs and Total-Etch Adhesive

**Published:** 2016-03

**Authors:** Farahnaz Sharafeddin, Mohammad Mehdi Choobineh

**Affiliations:** 1 Dept. of Operative Dentistry, Biomaterials Research Center, School of Dentistry, Shiraz University of Medical Sciences, Shiraz, Iran.; 2 Undergraduate Student, School of Dentistry, International Branch of Shiraz University of Medical Sciences, Shiraz, Iran.

**Keywords:** Self-etch Adhesive, Total-etch Adhesive, Nanofilled Composite, Shear Bond Strength, Conventional Glass-ionomer Cement

## Abstract

**Statement of the Problem:**

In the sandwich technique, the undesirable bond between the composite resin and glass-ionomer cement (GIc) is one of the most important factors which lead to the failure of restoration. Total-etch and self-etch adhesives may improve the bond strength based on their pH.

**Purpose:**

The purpose of this study was to evaluate the shear bond strength between the nanofilled composite resin and GIc using different adhesives.

**Materials and Method:**

In this experimental study, 40 specimens (6×6mm) in 4 groups (n=10) were prepared in acrylic mold. Each specimen contained conventional GI ChemFil Superior with a height of 3mm, bonded to Z350 composite resin with a height measured 3mm. In order to bond the composite to the GI, the following adhesives were used, respectively: A: mild Clearfil SE Bond self-etch (pH=2), B: intermediate OptiBond self-etch (pH=1.4), C: strong Adper Prompt L-Pop (pH=1), and D: Adper Single Bond 2 total-etch (pH=7.2). The shear bond strength was measured by using universal testing machine with a crosshead speed of 1mm/min. One-way ANOVA and Tukey’s test were used to analyze the data (*p*< 0.05).

**Results:**

The shear bond strength in group A was significantly higher than group B (*p*= 0.002), C (*p*< 0.001), and D (*p*< 0.001). Moreover, the shear bond strength of groups A and B (self-etch) was significantly different from group D (total-etch) (*p*< 0.001); and C (self-etch) with D (*p*= 0.024).

**Conclusion:**

The results of this study showed that applying the mild self-etch adhesive between the composite and the GIc results in stronger shear bond strength compared to intermediate and strong self-etch adhesives. Moreover, the self-etch adhesive increased the shear bond strength between composite resin and GIc more significantly than total-etch adhesive.

## Introduction

Laminate technique or sandwich restoration is one of the methods used in dental composite restoration,[1] in which two different materials namely glass-ionomer cement (GIc) and composite resin are used. In this technique, the GIc or resin-modified glass-ionomer cement (RMGIc) is placed between the dentin gingival margins and occlusal composite restoration.[2] The proper bond between GIc and resin composite is necessary for successful restoration. This method is mainly applied to benefit from both the physical and aesthetic properties of these materials. GIc presents two interesting features in restorations by bonding spontaneously to the dentin and releasing fluoride.[2] Some disadvantages of these materials include poor physical-mechanical properties and esthetics which can be compensated by the overlying composite resin.[3-4]

Etching the GIc is effective to obtain the favorable bond of composite.[5] Using 35% phosphoric acid as surface treatment of GIc may increase the shear bond strength of this cement to composite resin.[6]

The bond strength between the conventional GIc and composite resin is due to the porosity in the etched surface of GIc.[7] It has been found that in etching procedure, a 0.5 mm thickness of GIc and 20 seconds of etching is necessary to provide a proper bonding surface.[8]

In order to reinforce the bond strength between the GIc and composite resin, surface treatment with self-etch system has been suggested. Since the self-etch system has less technique sensitivity, it can mostly meet the dentists’ need for using sandwich technique.[9] The self-etch system can be either one-step or two-steps application procedure. Considering the invasion of self-etch adhesives, they are divided into strong, intermediate and mild versions.[10-11] It has been reported that higher acidity of the self-etch adhesive results in higher dentin demineralization. The strong self-etch adhesive has a pH of 1 or less. The self-etch with lower pH offers low bond strength, particularly in the dentin. The mild self-etch adhesive, however, generally has a pH of 2 and this low acidity causes a superficial demineralization, being less than 1 mm in dentin.[12-13]

Previous studies revealed the self-etch adhesive provided higher shear bond strength between the RMGIc and composite resin than other adhesives.[14-15] In a study, Mount showed that the changes in the pH of the adhesive affected the bond strength between the GIc and composite resin.[16] It is reported that applying self-etch adhesive on the surface of the GIc before using the composite resin improved the bond strength. It also decreased the clinical time because of the synchronous penetration of the adhesive resin along the self-etch process.[17]

Apparently, the bond strength between GIc and composite resin considerably affects the clinical success of esthetic restorations. Hence, the present study was designed to evaluate the shear bond strength of nanofilled composite bonded to conventional GIc by using self-etch adhesives with different pHs and total-etch adhesive. 

## Materials and Method

**Figure 1:**
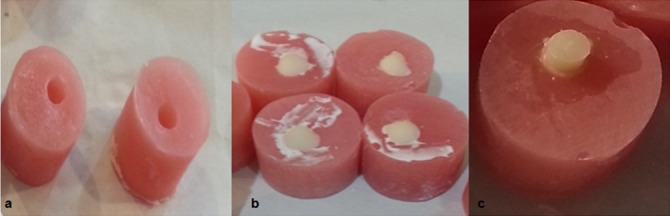
a: Preparation of acrylic mold. b: Glass-ionomer in acrylic mold. c: Resin composite on the surface of glass ionomer.

In this experimental study, 40 specimens of 6×6mm were prepared in 4 groups (n=10) in acrylic mold (2.5×2.5 cm). First, a hole (3mm height×6mm diameter) was created at the top of this acrylic cylinder by using bur #14. This hole was filled with ChemFil Superior GIc (Dentsply; Germany) with a proportion of 2:2 in powder and liquid according to the manufacturer’s instruction. The excess of GIc was removed by celluloid strip and glass slap in order to put the GIc and the acrylic molding at the same level. It was accurately checked for each specimen. After 7 minutes of initial setting of the GIc, the adhesive resin was applied on the surface of GIc (all according to manufacturer's instruction) (Figure 1). As represented in Table 1, the adhesives used in this experiment were self-etch strong Adper Prompt L-Pop (3M; ESPE), intermediate OptiBond (Kerr; Orange, CA, USA), mild Clearfil SE Bond (Kuraray; Tokyo, Japan) and total-etch adhesive Adper Single Bond 2 (3M; ESPE). Then, they were all light-cured by an LED light- cure device (Kerr Corp.; Orange, CA, USA) with an intensity of 1200 mW/cm2. The tip of the light-curing unit was placed 1 mm above the materials surface. Finally, Z350 composite (3M ESPE; USA) was applied on the GIc surface in two layers of 3×6mm (height × diameter).

**Table 1:** Bonding agents used in the study

**Groups**	**Adhesives**	**Manufacturer**	**Composition**	**PH**
A	Clearfil SE Bond	Kuraray Medical Inc, Tokyo, Japan	Primer E: HEMA, hydrophilic dimethacrylate, MDP(10-methacryloyloxydecyl dihydrogen phosphate), N, N-diethatol-p-toluidine, D,L-camphorquinone, water Adhesive E: Silanated colloidal silica, bisphenol A diglycidyl-methacrylate, HEMA, MDP, hydrophobic dimethacrylate, N,N-diethatol-p-toluidine, D,L-camphorquinone	2
B	OptiBond	SDS Kerr Orange, CA, USA	Water, ethyl alcohol, alkyl dimethacrylate resins, barium aluminoborosilicate glass, silicon dioxide, sodium hexafluorosilicate, stabilizers, and activators	1.4
C	Adper Prompt L-Pop	3M ESPE, St.Paul, USA	Liquid 1: methacrylate phosphoric esters, bis-GMA, camphorquinone, stabilizers, Liquid 2: water, HEMA, polyalkenoic acid, stabilizers	1
D	Adper Single Bond 2	3M ESPE, St. Paul, USA	Bis-GMA, HEMA, dimethacrylates, ethanol, water, novel photoinitiator system, methacrylate functional copolymer of polyacrylic and polyitaconic acids	7.2

In group A, the mild self-etch adhesive was applied on the GIc surface according to the manufacturer’s instruction by using a microbrush. Then, it was mildly air-dried and light-cured for 10 seconds. Finally, the composite was applied on the GIc surface in two layers of 3-mm high and cured for 40 seconds. In groups B and C, the procedure was the same as what was done in group A, except that intermediate self-etch adhesive, and strong self-etch adhesive were used instead, respectively. In group D, after applying the mixed GIc and 7 minutes of rest for initial setting (according to the manufacturer’s instruction), the surface was covered by 37% phosphoric acid for 15 seconds, and was then rinsed.[6] After that, the total-etch adhesive was applied to the GIc surface by a microbrush. Finally, the composite was added to the samples as in other groups.

**Figure 2:**
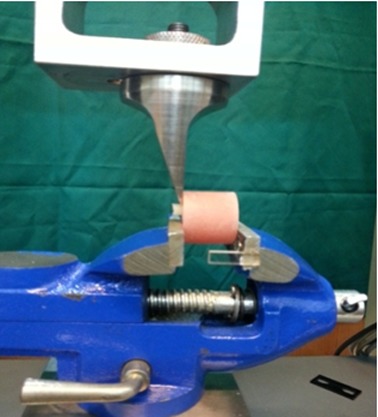
Testing the shear bond strength by using universal testing machine.

The samples were all stored in distilled water for 24 hours at room temperature. The shear bond strength was evaluated by the universal testing machine (Zwick/Roell Z020; Germany) at a crosshead speed of 1 mm/min (Figure 2).

The obtained data were analyzed by using SPSS software, version 20. One-way ANOVA and Tukey test were used to compare the mean shear bond strength among the groups. The significance level was set at 0.05. 

## Results

The mean±SD of shear bond strength of each group are presented in Table 2. The shear bond strength in group A (mild self-etch adhesive) was significantly higher than group B (*p*= 0.002), C (*p*< 0.001) and D (*p< *0.001). The maximum and minimum shear bond strength was obtained respectively in Group A with the mean of 7.77 MPa and group D (total-etch adhesive) with the mean of 3.45 MPa.

**Table 2:** The mean shear bond strength of the study groups calculated by using One-way ANOVA

**Groups**	**Adhesive Agents**	**Mean±SD**	**P value**
A(CLEARFIL™ SE BOND)	Mild self- etch bonding	7.77±0.82	<0.001
B(OptiBond®)	Intermediate self-etch bonding	6.04± 0.71	
C(Adper™ Prompt™ L-Pop™)	Strong self-etch bonding	4.71±1.34	
D(Adper™ Single Bond 2)	Total-etch bonding	3.45±0.78	

**Figure 3:**
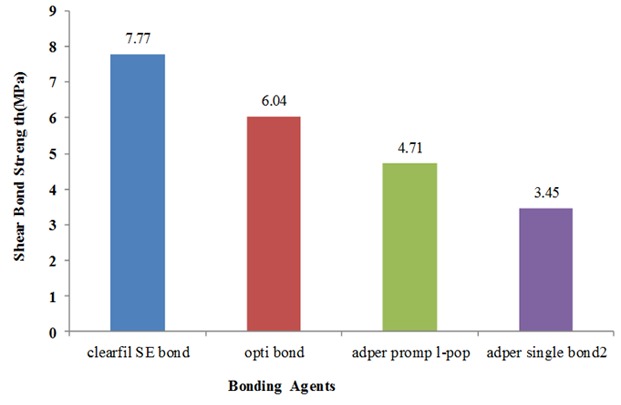
The shear bond strength of the tested groups

Comparing the groups by Tukey’s test (Table 3), a statistically significant difference was detected among the shear bond strength of the groups. According to the results of Tukey’s test, there was a statistical difference between the shear bond strength of group A and B (*p*= 0.002), and between group A and C (*p*< 0.001). A comparison of group B and C showed that the values obtained from them are significantly different (*p*= 0.002). Likewise, comparing groups A and B with group D (*p*< 0.001), and group C with D (*p*= 0.024) showed their shear bond strength were significantly different. Figure 3 shows that the shear bond strength of the four tested groups.

**Table 3:** Pairwise comparison by Tukey test

**Groups**	**Groups**	**P value**
Clearfil SE bond	OptiBond Adper Prompt L-Pop Adper Single Bond 2	.002 <0.001 <0.001
OptiBond	Adper Prompt L-Pop Adper Single Bond 2	.012 <0.001
Adper Prompt L-Pop	Adper Single Bond 2	.024

## Discussion

Proper bond strength between resin composite and GIc is necessary for the success of sandwich technique. Applying self-etch adhesive over GIc creates a stronger bond of composite resin to GIc compared with total-etch adhesive.[15] The total-etch system needs two separate phases of rinsing and drying and has also a higher technique sensitivity.[18-19] Moreover, the GIc surface may become weak during the drying phase.[20]

Owing to the acidic monomer in its composition, the self-etch adhesive does not need the etching phase, rinsing and drying.[21] Self-etch adhesives are categorized into groups of mild, intermediate and strong based on their pH level and etching potential.[22] Thus, their ability in creating a bond between the composite and GIc may be different.

Our study showed that the mild self-etch adhesive (Clearfil SE Bond) yields a higher shear bond strength between the ChemFil GIc and Z350 composite compared with the intermediate (OptiBond) and strong self-etch adhesive (Adper Prompt L-Pop). Similar results were achieved by Kandaswamy *et al.* who reported that the mild self-etch bonding provided higher shear bond strength.[23] This might be due to the lower acidity of the mild self-etch adhesive compared with the strong and intermediate self-etch adhesives. According to organic chemistry, when a weak acid invades something, it induces a minimum excitation in the ions, and hence the salt crumps formation will be minimal.[24] Cations such as Ca2+ and Na+ that are not excited and are present in large amounts for effective interaction, especially in a conductive reaction medium like GIc, instigate strong ionic reaction with the bonding agents.[15, 25] It seems that the lower acidity of mild self-etch adhesive leads to the higher shear bond strength. 

Additionally, in our research, the strong self-etch adhesive system in group C (pH=1) had a lower shear bond strength compared with the other two groups of self-etch bonding (groups A and B). Some previous studies showed that using self-etch adhesive with a lower pH (1-0.8) created lower shear bond strength.[23, 26-27]Stronger acid neutralizes more cations, resulting in salt crumps formation. Therefore, the structure of the GIc is weakened and fragile, thus consequently the bond will be weakened.[23, 25]

This research also found that self-etch adhesives improved the bond between the composite and conventional GIc compared with the total-etch adhesive. Arora *et al.* reported that the self-etch adhesive caused a stronger shear bond between the composite and RMGIc.[14] Similar result was achieved in a study conducted by Chandak *et al.* on the same issue.[28] Another study also showed that using self-etch adhesive on the surface of RMGIc had the potential of creating a better bond strength with the resin composite.[15]This might be due to the acidic pH of self-etch adhesive. The acidic characteristic of self-etch adhesive causes superficial dissolution of GIc and consequently improves the bond between composite resin and GIc.[29]Etching the surface of GIc with 37% phosphoric acid leads to dissolution of the lower layers of GIc matrix and therefore, would decrease the cohesive strength of the GIc which subsequently can affect the bond strength of the composite and GIc adversely.[15, 30] The porosity created on the GIc surface due to the phosphoric acid is different from that caused by self-etch adhesive. Superficial destruction by means of acid-etching leads to an undesirable surface bond with the composite. It seems that application of an acid with a similar acidity of the self-etch adhesive helps creating better shear bond strength.

On the other hand, self-etch adhesive has a lower viscosity compared with the total-etch adhesive.[31] In a research, Mount found that bonding with a lower viscosity caused low contact angle on the surface; thus, it improved the wettability and strengthened the bonding of resin composite and GIc.[16] It seems that low viscosity of self-etch adhesive has more potential of wettability compared with the total-etch adhesive; so it provides greater shear bond strength between the composite and GIc.

Previous studies on self-etch adhesives showed that this system bonds with the calcium in the structure of the teeth;[5] therefore, it can possibly bond with the calcium in the structure of GI and create a higher shear bond strength compared with the total-etch adhesive.

Overall, further studies are recommended to examine and evaluate the effect of different generations and the application of bonding with different pHs on the bond strength between the composite and light GI. 

## Conclusion

Concerning the limitations of this study, it can be concluded that using the mild self-etch adhesive (Clearfil SE Bond) between the resin composite and GIc increases the shear bond strength compared with the strong (Adper Prompt L-Pop) and intermediate (OptiBond) self-etch adhesive. Moreover, using self-etch adhesive between the GIc and composite resin creates a higher shear bond strength compared with total-etch adhesive (Adper Single Bond 2). 
